# A comparative study of logistic regression based machine learning techniques for prediction of early virological suppression in antiretroviral initiating HIV patients

**DOI:** 10.1186/s12911-018-0659-x

**Published:** 2018-09-04

**Authors:** Kuteesa R. Bisaso, Susan A. Karungi, Agnes Kiragga, Jackson K. Mukonzo, Barbara Castelnuovo

**Affiliations:** 1 Makerere University Infectious Diseases Institute, P.O. Box 7072, Kampala, Uganda; 2 Department of Pharmacology and Therapeutics, Makerere University College of Health Sciences, Kampala, Uganda; 3 Breakthrough Analytics Ltd, Kampala, Uganda

**Keywords:** Prediction, Viral suppression, Machine learning, Multitask temporal logistic regression, Patient specific survival prediction, Logistic regression, L2-regularization

## Abstract

**Background:**

Treatment with effective antiretroviral therapy (ART) lowers morbidity and mortality among HIV positive individuals. Effective highly active antiretroviral therapy (HAART) should lead to undetectable viral load within 6 months of initiation of therapy. Failure to achieve and maintain viral suppression may lead to development of resistance and increase the risk of viral transmission. In this paper three logistic regression based machine learning approaches are developed to predict early virological outcomes using easily measurable baseline demographic and clinical variables (age, body weight, sex, TB disease status, ART regimen, viral load, CD4 count). The predictive performance and generalizability of the approaches are compared.

**Methods:**

The multitask temporal logistic regression (MTLR), patient specific survival prediction (PSSP) and simple logistic regression (SLR) models were developed and validated using the IDI research cohort data and predictive performance tested on an external dataset from the EFV cohort. The model calibration and discrimination plots, discriminatory measures (AUROC, F1) and overall predictive performance (brier score) were assessed.

**Results:**

The MTLR model outperformed the PSSP and SLR models in terms of goodness of fit (RMSE = 0.053, 0.1, and 0.14 respectively), discrimination (AUROC = 0.92, 0.75 and 0.53 respectively) and general predictive performance (Brier score= 0.08, 0.19, 0.11 respectively). The predictive importance of variables varied with time after initiation of ART. The final MTLR model accurately (accuracy = 92.9%) predicted outcomes in the external (EFV cohort) dataset with satisfactory discrimination (0.878) and a low (6.9%) false positive rate.

**Conclusion:**

Multitask Logistic regression based models are capable of accurately predicting early virological suppression using readily available baseline demographic and clinical variables and could be used to derive a risk score for use in resource limited settings.

## Background

Treatment with effective ART decreases morbidity and mortality among HIV positive individuals [1, 2]. Effective antiretroviral therapy (ART) should lead to undetectable viral load within 6 months of initiation of therapy [3]. Achievement of early viral suppression (suppression by 24 weeks) predicts long term treatment success as measured by virological suppression, CD4+ cell count increase and reduction in mortality [4, 5]. However in sub-Saharan Africa, more than 24% of patients receiving first line ART have virological failure within 1 year of initiation of therapy [6, 7]. Furthermore, treatment failure and subsequent switching of therapy from first line to second line ART was reported to occur as early as 6 and 7 months respectively, after ART initiation in resource limited settings [8, 9]. Failure to achieve and maintain viral suppression may lead to development of resistance and increase the risk of viral transmission [6, 10, 11].

Attainment of early virological suppression depends on a number of factors including choice of initial ART regimen especially in ART naïve patients, ART adherence, comorbidities, and inter-individual variability in drug pharmacokinetics, demographic and genetic factors and drug resistance, baseline viral load and CD4 count [12–19]. Leveraging the knowledge of a combination of all or some of these factors through rapid risk calculation to predict early viral outcomes in individual patients before initiation of ART would enhance clinical decision making and prevent adverse outcomes of treatment failure and the costs associated with switching to second line ART [20].

Machine learning models have been developed and used to predict virological response to ART. However, the use of such models to guide therapeutic decision making may be limited by two major reasons. Many of these models heavily rely on viralogical resistance genotype data which may not be available in resource limited settings [21–23]. Those that avoide genotype data make use of relatively complex classifiers such as random forests(RF) or artificial neural networks (ANN) as the backbone of on-line prediction tools [20, 24–27]. Such tools and methods are not easily interpretable by medical providers and are inaccessible in resource limited settings where computing facilities may not be available. Logistic regression is popular among medical practitioners owing to its interpretability and ease of application without need for a computer. Therefore, logistic regression based machine learning may solve the above mentioned limitations of the available virological response prediction tools. The purpose of this study was to assess the performance of 3 logistic regression based machine learning methods at predicting early virological failure in HIV patients initiating ART.

## Methods

### Patient cohorts

Data from two independent cohorts was used in this analysis. The Infectious Diseases Institute (IDI) cohort data was used for training the prediction model and testing its generalizability while data from the efavirenz (EFV) cohort was used to test the model’s ability to predict outside the studied population (transportability).

This IDI cohort data obtained from the integrated clinic enterprise application (ICEA) database implemented and maintained at IDI [28]. The database is regularly validated for quality, completeness and discrepancies. The data consists of 559 consecutive HIV patients enrolled between April 2004 and April 2005. Upon recruitment, patients were initiated on one of 3 ART regimens namely stavudine/lamivudine/nevirapine (30/300/200 mg) or (40/300/200 mg) and Efavirenz /Zidovudine/Lamivudine (600/150/300 mg). Patients were followed up every 6 months but intermediate visits occurred for some patients. Patient information was collected on all visits and included demographic data, previous and current opportunistic infections, non-HIV related clinical events, WHO stage, vital signs, ART regimen, physical examination results, adherence to ART, ART toxicity, ART substitution reasons, complete blood count, liver and renal function tests, CD4 count, HIV viral load, death and the cause of death. The cohort is still undergoing observation and details about the cohort study procedure have been reported before [29–31]. The observational study was approved by the institutional review board and Uganda National Council of Science and Technology (UNCST).

The EFV cohort data consisted of a cohort that was recruited for an Efavirenz dose optimization study [32]. The data consists of 262 ART naïve HIV/AIDS patients treated for HIV with standard dose Efavirenz /Zidovudine/Lamivudine (600/150/300 mg). These patients were recruited from Mulago National referral hospital, kampala (*n* = 155), Butabika hospital, kampala (*n* = 60) and Bwera hospital, kasese (*n* = 47) in the years 2008 and 2009. One hundred and fifty eight of those were TB co-infected at the time of initiation of ART. Only 235 patients in this data had viral load counts collected in the first 6 months of ART. Baseline demographic characteristics (age, weight, sex, TB disease status) as well as CD4 count and viral loads were collected in these patients Follow-up visits occurred on days 3, 56, 84, 112, 140, 148 and 168. Each participant provided at least 2 viral load count measures. The study was approved by the institutional review boards and UNCST. Details about the data have been published before [32].

### The machine learning algorithms

Three logistic regression based modelling approaches were used to model the longitudinal data. These included; Simple logistic regression (SLR), multitask temporal logistic regression (MTLR) and patient specific survival prediction modelling (PSSP).

#### Simple logistic regression

In this approach, all data was aggregated together as if the outcome occurred at the same time point (6 months). The outcome variable was set to 1 if viral suppression was achieved or 0 if not achieve at 6 months of ART. Baseline predictors were used to predict the outcome using logistic regression.If*y*_*i*_ and x_i_ are the observation and its corresponding vector of predictors respectively, such that *y*∈[0, 1] and *θ* is a vector of coefficients, the probability of virological suppression is given by

1 $$ {h}_i=P\left({y}_i=1|{x}_i,\theta \right)=\frac{1}{1+{e}^{-{\theta}^T{x}_i}} $$

L2-regularization was applied to the model to reduce overfitting. This was accomplished by optimizing the following cost function.

2 $$ \sum \limits_{i-1}^N\left({y}_i\log \left({h}_i\right)+\left(1-{y}_i\right)\log \left(1-{h}_{i,}\right)\right)+{\lambda}_1{\left\Vert \theta \right\Vert}_2^2 $$

The hyperparameters λ_1_ controls overfitting and N is the total number of individuals in the training dataset.

#### Multitask temporal logistic regression (MTLR)

Each clinic visitation day was assumed to be a unique learning task for which a logistic regression classification model was trained (fitted) and the task specific parameter (coefficients) and probability of virological suppression learned (estimated). Thus for any task *t* in *[1,2...,M],* if*y*_*i,t*_ and x_i,t_ are the observation and its corresponding feature vector respectively, such that *y*∈[0, 1] and *θ*_*t*_ is a vector of task specific coefficients, the probability of virological suppression is given by

3 $$ {h}_{i,t}=P\left({y}_{i,t}=1|{x}_{i,t},{\theta}_t\right)=\frac{1}{1+{e}^{-{\theta}_t^T{x}_{i,t}}} $$

For each task, overfitting was reduced by explicitly controlling the complexity of the model using L2 regularization as described later. Additionally, the similarity between tasks was leveraged without concealing their uniqueness by applying the multitask learning approach. Specifically, all tasks were learned jointly such that the temporal relation between tasks was enforced. This was accomplished by optimizing the following cost function.

4 $$ \sum \limits_{t=1}^M\left[\sum \limits_{i=1}^{N_t}\left(\ {y}_{i,t}\log \left({h}_{\mathrm{i},\mathrm{t}}\right)+\left(1-{y}_{i,t}\right)\log \left(1-{h}_{i,t}\right)\right)+{\lambda}_1{\left\Vert {\theta}_t\right\Vert}_2^2\right]+{\lambda}_2\sum \limits_{t=1}^{M-1}{\left\Vert {\theta}_{t+1}-{\theta}_t\right\Vert}_2^2 $$

The first term is likelihood of suppression across all tasks, the second term limits the generalization error via the L2- regularization and the third term enforces the temporal smoothness on weights from adjacent tasks. The hyperparameters λ_1_and λ_2_ control overfitting and temporal smoothness, respectively.

#### Patient specific survival prediction modeling (PSSP)

In this approach, we formulated the problem as a survival one for each patient using the method developed and described by Yu et al. [33]. The aim was to predict whether or not suppression occurs within 168 days and the time at which it occurs for each patient. The dataset was restructured to include only the 4 most commonly shared observation times namely t = {0, 84, 98, 168}, also referred to as tasks, *t = {1,..,M},* where *M = 4*. Patient outcomes, *y*_*t*_∈[0, 1] were recorded for each time point, for each patient, capturing the dependence between observations. Thus, if ***S*** is the time point at which undetectable viral load is first recorded for the n^th^ patient, then at all t < S, y_i_ = 0 while at all t ≥ S, y_i_ = 1. The elements of the sequence *y = (y*_*1*_*, y*_*2*_*,…,y*_*M*_*)* of outcomes over all four time points were encoded as y_t,n_^(s)^for the value at time t, where s is the survival time in the sequence. For our 4 time points, there are 5 possible sequences, including a sequence of all 0 s. The logistic regression method was extended to model the probability of observing the survival status sequence for the n^th^ patient as follows:

5 $$ {h}_i=p\left(Y=\left({y}_1,\dots {y}_T\right)|{x}_n,\Theta \right)=\frac{e^{\left({\sum}_{j=1}^T{y}_j\left({\theta}_j^T{x}_n\right)\right)}}{\sum_{k=0}^M{e}^{f\left({x}_n,k,\Theta \right)}} $$

Where Θ is the set of all parameter vectors *(θ*_*1*_*,..,θ*_*M*_*)* and $f\left(x,k,\Theta \right)=\sum \limits_{i=k+1}^M\left({\theta}_i^Tx\right)$ for *0 < k < M* with viral load becoming undetectable (y = 1) in the interval [t_k_, t_k + 1_]. In order to predict patient specific survival probabilities and times, we optimize the following cost function:

6 $$ \sum \limits_{n=1}^N\left[\sum \limits_{t=1}^M{y}_{t,n}^{(s)}\log \left({\theta}_t^T{x}_n\right)-\log \sum \limits_{k=0}^M{e}^{f\left({x}_n,k,\Theta \right)}\right]+{\lambda}_1\sum \limits_{t=1}^M{\left\Vert {\theta}_t\right\Vert}_2^2+{\lambda}_2\sum \limits_{t=1}^{M-1}{\left\Vert {\theta}_{t+1}-{\theta}_t\right\Vert}_2^2 $$

The first term is the log-likelihood of observing a sequence given parameters θ = [θ_1_,..,θ_M_] and baseline predictor variables, *x*for all *N* patients. The second term is the L2 regularizer that prevents overfitting and the third term is a regularizer that enforces temporal smoothness on parameters from adjacent observation time points. The hyperparameters λ_1_ and λ_2_ control overfitting and temporal smoothness, respectively.

### Data preparation and model building

#### Data preparation

The outcome of interest was viral suppression. This was coded in each row (corresponding to an observation) as 0 or 1 depending on whether the viral load count was above or below 400 copies /ml respectively. The choice of viral load cut-off was based on the lower limit of quantification of the assay (400 copies /ml) at the time of recruitment. The EFV cohort viral load measurements had a lower limit of quantification of 40 copies per ml. However, for this analysis, a cut off of 400 copies/ ml was applied because it encompasses both datasets. The proportion of undetectable viral load observations in the IDI cohort and EFV cohort datasets was 0.47 and 0.69 respectively.

The observation time (clinic visit) was recorded as days after initiation of ART, corresponding to follow-up visits. Patient data up to day 180 (corresponding to 6 calendar months) and day 168 in the IDI and EFV cohorts respectively was used in this analysis. This is because early virological suppression is expected to have occurred by this time if treatment and patient management are effective.

#### Predictor variables

The predictor variables (features) in the data included sex, baseline age and body weight, TB disease status, ART regimen, baseline CD4 count and viral load (VL) count. Sex coded as 0 or 1 for female and male participants respectively. TB disease status was coded as 0 or 1 depending on whether the participant had been diagnosed at the start of ART with or without TB respectively. ART therapy was also coded with numbers 1 to 3 corresponding to the regimen a patient was initiated on. Age, body weight, CD4, viral load count were left as continuous variables. All these features have been previously reported in literature to have a relationship with virological outcomes [15, 34]. Model training and testing utilized the IDI cohort data.

#### Data splitting

The IDI cohort dataset was randomly split into training and testing sets in the ratio 2:1 based on individual ID numbers. The training dataset consisted of 322 individuals (765 labelled examples) while the test dataset consisted of 162 individuals (380 labelled examples). The training dataset was used to train the model and learn the feature coefficient (weights). The test dataset was used to assess the performance of the model in predicting outcomes in a previously unseen dataset from a reasonably related population. This is also known as model generalizability testing. Care was taken in the choice of the splitting ration to ensure that the training examples were sufficient and the testing dataset had a minimum of 100 positive and negative outcomes each [35, 36].

#### Hyperparameters optimization

An exhaustive search for the optimal L2-regularization and temporal smoothing parameters (λ_1_ and λ_2_) from a set of 302 pre-specified candidates ranging from 0 to 1000 was done using the grid search method. The combination of λs that maximized the model’s predictive performance on the training dataset was selected as follows. For each of the candidate hyperparameter combination, a 5- fold cross validation was carried out on the training dataset. The training dataset was randomly split into 5 equal parts. Four parts (80% of the data) were used to learn the model coefficients. The fifth part (20%) was used to compute the area under the receiver operator characteristics curve (AUROC). The operation was repeated until each of the five parts had been used for testing. The mean AUROC over the 5 runs was computed. The hyperparameter combination corresponding to the highest mean AUROC was selected and used for model training.

#### Cost function optimization

The cost functions were optimized using the BFGS (for MTLR and SLR) and Nelder-Mead (for PSSP) algorithms as implemented in the optim library in R software [37–39]. At least 10 retries with different sets of starting parameters were used to ensure convergence and stability of the final coefficient estimates. Bootstrap analysis, using 1000 bootstrap replicates was used to obtain the bootstrap mean, median and the 95% confidence intervals for the parameter estimates using the training dataset [40].

### Model validation

The goodness of fit (reliability) plot depicting agreement between the observed proportion of viral suppression and predicted probability of virological suppression were generated for each model [41]. In this plot the range of predicted probabilities was discretized into 20 intervals. The mean predicted probability and the associated observed proportion of viral suppression in each interval were calculated and plotted. The points should be near to the diagonal if the model is well calibrated, otherwise the model would be misspecified [42–44]. A corresponding sharpness diagram was plotted to show the distribution of the different probability categories used to generate the reliability plot. The root mean squared error (RMSE) with respect to the identity line was also calculated.

The calculated probabilities were used to assess the overall predictive performance of the models by calculating the means squared error (MSE), also known as the brier score [45]. Since the outcome prevalence in the test datasets used for the MTLR and PSSP was 0.47, a brier score of less than 0.245 was considered as satisfactory predictive performance. For the SLR model, the outcome prevalence in the dataset was 0.115 thus a brier score less than 0.102 was considered satisfactory [41].

The model’s discriminative ability was assessed by generating a receiver operator characteristics curve and the corresponding c-statistic (AUROC), and the precision-recall curve and the corresponding area under the precision recall curve (AUPRC) using the non-parametric method [46]. A c-statistic is a measure of the ability of the model to correctly classify those with and without the outcome. C-statistic values of 0.5–0.7,0.7-0.79,0.8–0.89 and > 0.9 were considered, poor, moderate, good and excellent predictions respectively [47]. An AUPRC value above 0.47 was considered satisfactory. The F1 score, which is the harmonic mean of precision and recall, was also calculated. The closer the F1 score to 1 the higher the discriminative ability of the model while values close to 0 meant poor discrimination [48].

The Youden indices (J-statistic) of each model was obtained by searching among plausible values of the predicted probability of outcome for which the sum of sensitivity and specificity was a maximum [49]. For any task, if the patient’s predicted probability was above the obtained J-statistic, viral suppression was predicted to occur therefore the J-statistic was considered the decision boundary (cut-point) between low and high probability patients [50, 51].

Using the cut-point, the performance of the models outside the studied population, setting and period, also known as temporo-spatial transportability was assessed on the EFV cohort dataset, to ensure practical applicability of the model [52]. The shared tasks between the IDI and EFV datasets were day 1, 84, 112, 140 and 168 and model transportability was tested only on these tasks.

The models were used to predict probability of suppression in the EFV dataset. The prediction accuracy, sensitivity, selectivity, positive negative predictive value and positive predictive value were generated for each model.

## Results

The distribution of variables between the two cohorts was similar as shown in Table 1 below.

**Table 1:** distribution of variables in the EFV and IDI cohort datasets

Variables	IDI cohort (*n* = 484)	EFV cohort (*n* = 233)
WT/kg (median [IQR])	55.0 [48.0–61.0]	51.0 [47.0–58.0]
AGE/ years (median [IQR])	35.0 [30.0–41.0]	33.0 [30.0–40.0]
CD4 / cell per ml (median [IQR])	100.0 [29.7–166.0]	109.0 [46.0–179]
VL*1000 copies per ml (median [IQR])	349 [116.5–595.2]	123.7 [42.7–253.7]
SEX (male %)	30.4	44.6
TB / %(n)	7	57.5
REGIMEN 1 d4T/3TC/NVP-30 (%)	49.5	0
REGIMEN 2 d4T/3TC/NVP-40 (%)	24.7	0
REGIMEN 3 AZT/3TC/EFV (%)	25.6	100

**Fig. 1:**
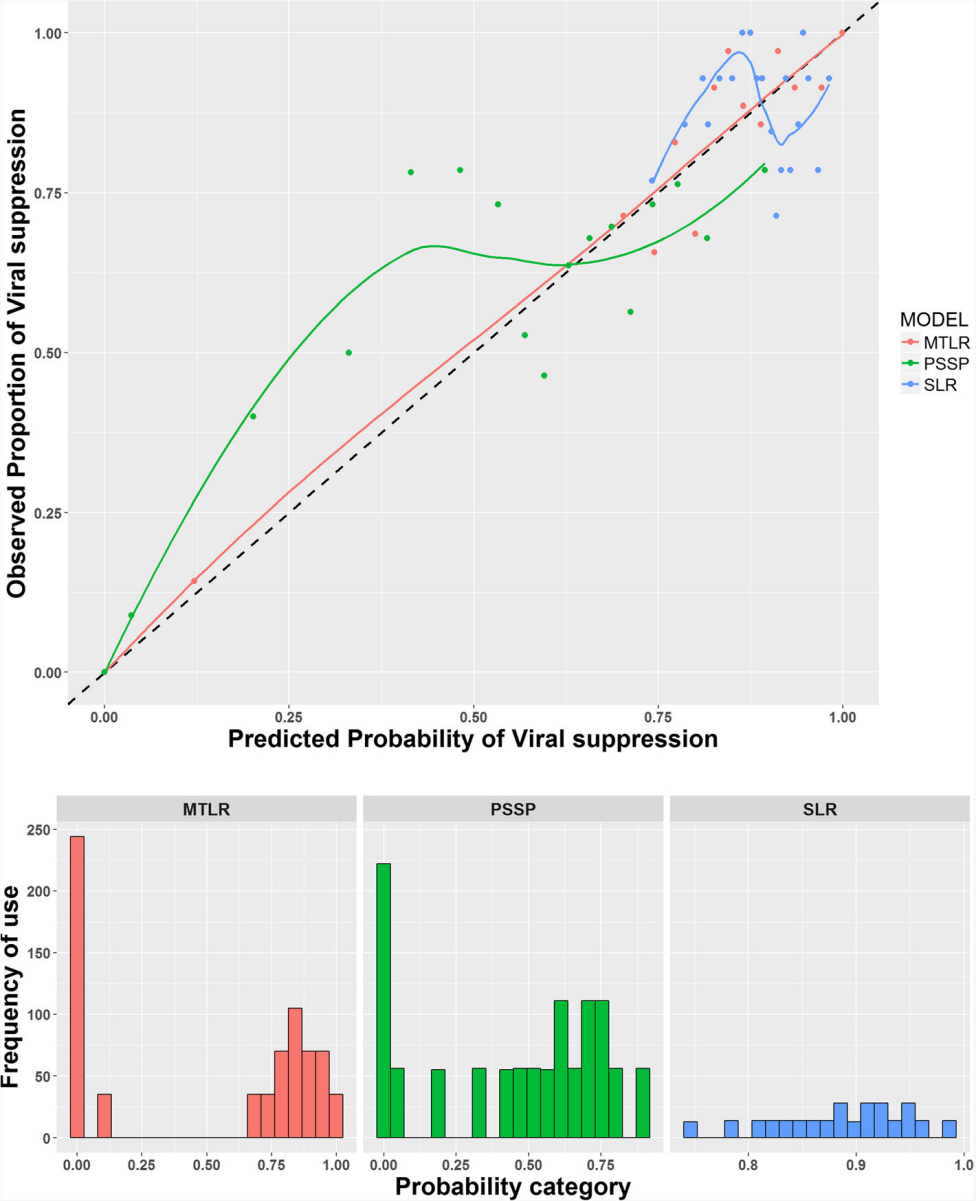
Reliability plots showing model calibration. The lower plot is the sharpness diagram showing the distribution of probability categories used to generate the reliability plot

The MTLR model adequately fit the data, implying good model calibration. The PSSP and SLR models showed poor fit to the training set, implying misspecification and poor reliability (see Fig. 1). The RMSEs with respect to the identity line were 0.053, 0.100 and 0.143 for the MTLR, PSSP and SLR models respectively.

The MTLR and PSSP models showed adequate overall predictive performance with Brier scores less than 0.245 as shown in Table 2. The SLR model did not show adequate predictive performance since it had a brier score higher than the maximum score of 0.102 (outcome prevalence in the dataset was 0.115).

**Table 2:** The 5 fold cross validated model discriminative characteristics and general predictive performance

MODEL	AUROC(SE)	AUPRC	F1	% ACCURACY	% TP	% TN	% FP	% FN	BRIER
MTLR	0.9204 (0.0186)	0.8706	0.9194	93.76	50.66	43.14	5.98	0.24	0.0814
PSSP	0.75 (0.027)	0.6584	0.7684	81.4	46.52	34.86	18.12	0.5	0.1974
SLR	0.538 (0.1042)	0.8752	0.937	57.94	49.54	8.4	3.58	38.44	0.1072

*TP* True positive, *TN* True negatives, *FP* false positives, *FN* False negatives

**Fig. 2:**
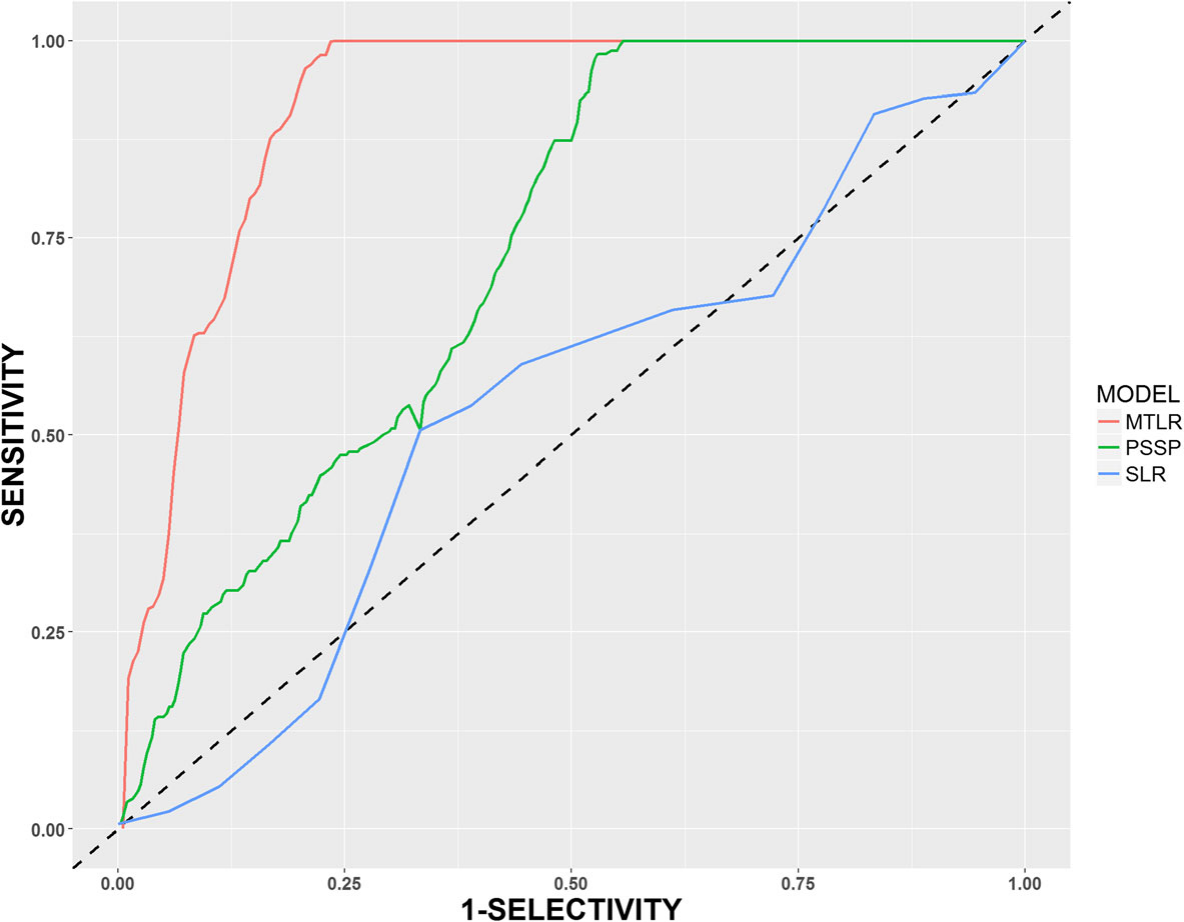
A receiver operator curve (ROC) and precision recall curve (PRC) showing the model discrimination abilities of outcomes in the IDI cohort

The MTLR, PSSP and SLR models showed excellent, moderate and poor discriminative abilities respectively with respect to AUROC as shown in Table 2 and depicted in Fig. 2.

**Fig. 3:**
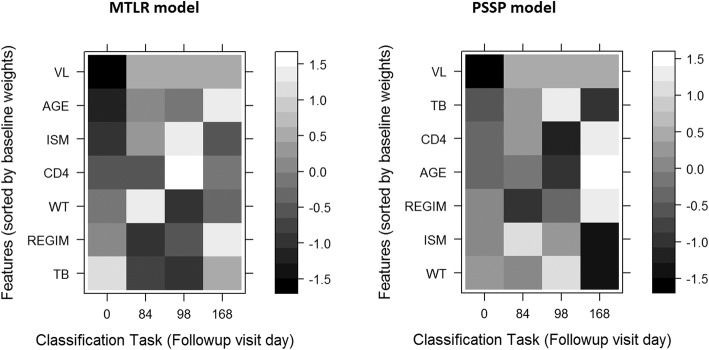
A heatmap showing changes in Feature importance with time after initiation of ART in the MTLR and PSSP models

Both the MTLR and PSSP models predicted viral suppression in the EFV cohort with adequate accuracy and discrimination as shown in Table 3 below and Fig. 3 below. The SLR model performed worse than random guessing in terms of prediction of discriminative performance on the EFV cohort.

**Table 3:** Discrimination and prediction accuracy of viral suppression in the EFV cohort by all models

MODEL	AUROC	AUPRC	F1	% Accuracy	% TP	% TN	% FP	% FN
MTLR	0.878 (0.016)	0.892	0.93	92.9	66.1	26.8	6.9	0.2
PSSP	0.824 (0.02)	0.817	0.921	92.3	66.3	26.0	7.7	0
SLR	0.497 (0.09)	0.938	0.971	24.6	21.5	3.1	2.6	72.8

Figure 3 shows variation of feature importance with time in MTLR model which was the best performing model. The change in appearance of each column compared to the first column indicates a change in the relative importance of the feature over time.

## Discussion

In this study, three modelling methods were developed to predict early virological outcomes in patients initiating ART, using their demographic and clinical data and their performance was compared.The machine learning approach to development and validation of these models was chosen to maximize the model prediction accuracy and generalizability since only a limited number of variables were used [53, 54]. Logistic regression based methods were employed because of the popularity of logistic regression among medical practitioners owing to its interpretability of parameters and ease of application. Prediction on external data was improved by penalizing the model coefficients using L2 regularization. L2 regularization was chosen over other regularization methods so as to retain all the selected features in the models but penalize their weights based on their contribution towards the overall predictive performance of the model. Nevertheless, the resultant coefficients cannot be used to infer associations because they are biased to maximize prediction [54].

The multitask learning approach employed in the MLTR and PSSP models captures the relatedness in the outcomes on the different follow-up visits, while retaining the peculiarities of the different outcomes [55]. The PSSP model combines logistic regression models at each task in a temporally dependent manner to form a survival function capable of predicting patient specific survival. On the other hand, the MTLR model does not enforce any dependency between logistic regression models at each task and therefore is a task specific classifier.In both models, temporal smoothness was enforced by regularization which reduced overfitting for the under-sampled tasks, improved prediction accuracy of all tasks and led to better overall generalizability of the model than that of the SLR model. The better predictive performance of the MTLR as compared to the PSSP model could imply absence of dependency between tasks in the data. In otherwords, viralogic status at any time point does not depend on and can not be infered from that at another time point.

The multitask models were able to capture the temporal structures of the outcomes in the data thus enabling the studying the temporal dynamics of the features as depicted in Fig. 3 [56, 57]. The normalized weights of these variables exhibited temporal variation. This implies the relative importance of variables changes over time. This might also explains why the SLR model which allows only a single weight per variable without accounting for temporal variation in feature importance did not fit the data as well as the multitask approaches.

Whereas the MTLR model was the best performing model in all aspects including prediction in the external EFV dataset, the predictive performance of the PSSP model was higher in the external EFV dataset than in the IDI dataset. It was not immediately clear why this was the case.The MTLR model was chosen over the PSSP and SLR models based on goodness of fit plots. Adequate goodness of fit results in model reliability while poor goodness of fit may imply model miss-specification which might affect reproducibility of the model’s predictive performance [42–44]. Basing on the goodness of fit plots, the MTLR model is likely to be more reliable than the other two, and was thus chosen as the final model for the subsequent analyses.

The final model was used to develop a risk score that stratifies patients into low and high risk of early virological failure using the Youden index as the cut-off point. The score had good prediction accuracy (92.9%) and satisfactory discriminatory performance (87.8%) in an external dataset from another cohort that is different in geographical location and year of recruitment, from the one used to train and validate the model. This implies that the model is applicable across geographical boundaries and is temporally consistent. The cut-off point was varied to maximize specificity so as to limit the number of false positives. False positive misclassification implies that some patients with virological failure may be missed and we wanted to avoid this. However, at maximum specificity the percentage of false positives (~ 7%) was similar to that at the specificity of the selected cut-off point, albeit with worse prediction accuracy and an increase in false negatives. False negative classification implies that some patients with viral suppression are misclassified as having virological failure. This can be costly in terms of confirmatory virological testing and clinical monitoring and choice of alternative ART regimens, which could strain the system. With the current cut-off point no false negatives were reported in both the test and external datasets, therefore we kept this cut-off point for further practical application.

The model has other predictive and practical advantages in resource limited settings. In these settings, absence of routine monitoring of viral load, pharmacogenetic and drug resistance mutation testing to guide choice of therapy pose a great challenge [58, 59]. In addition, health care system challenges affect accuratediagnosis, patient monitoring and provision of care, making it difficult to identify patients at risk of early virological failure [60]. Therefore this model could guide individualized clinical decisions such as choice of first line ART and clinical (virological and immunological) monitoring. The risk score can readily be calculated by hand.

## Conclusion

Three logistic regression based models were developed to predict early virological suppression using 7 baseline demographic and clinical variables. The multitask temporal logistic regression (MTLR) model outperformed the other models in all aspects, demonstrating adequate calibration properties and excellent classification and general predictive performance. The multitask models outperformed the simple logistic regression model. Logistic regression based models are capable of accurately predicting early virological suppression using readily available baseline demographic and clinical variables.

## Abbreviations

ART: Antiretroviral Therapy
AUPRC: Area Under Precision Recall Curve
AUROC: Area Under the Receiver Operating Curve
BFGS: Broyden-Fletcher-Goldfarb-Shanno algorithm
EFV: Efavirenz
HAART: Highly Active Antiretroviral Therapy
HIV: Human Imunodeficiency Virus
ICEA: Integrated Clinic Enterprise Application
IDI: Infectious Diseases Institute
MTLR: Multitask Temporal Logistic Regression
PSSP: Patient Specific Survival Prediction
ROC: Receiver Operating Characteristics
SLR: Simple Logistic Regression
UNCST: Uganda National Council of Science and Technology
VL: Viral Load

## References

[1] Bendavid E, Holmes CB, Bhattacharya J, Miller G (2012). HIV development assistance and adult mortality in Africa. JAMA.

[2] Palella FJ, Delaney KM, Moorman AC, Loveless MO, Fuhrer J, Satten GA (1998). Declining morbidity and mortality among patients with advanced human immunodeficiency virus infection. N Engl J Med.

[3] Günthard HF, Aberg JA, Eron JJ, Hoy JF, Telenti A, Benson CA (2014). Antiretroviral treatment of adult HIV infection: 2014 recommendations of the international antiviral society–USA panel. JAMA.

[4] Soe AN, Phonrat B, Tansuphasawadikul S, Boonpok L, Tepsupa S, Japrasert C (2010). Early viral suppression predicting long-term treatment success among HIV patients commencing NNRTI-based antiretroviral therapy. Journal of Antivirals & Antiretrovirals.

[5] Lohse N, Kronborg G, Gerstoft J, Larsen CS, Pedersen G, Sorensen HT, et al. Virological control during the first 6–18 months after initiating highly active antiretroviral therapy as a predictor for outcome in HIV-infected patients: a Danish, population-based, 6-year follow-up study. Clin Infect Dis. 2006;42(1):136–44. 10.1086/498515 16323104

[6] Barth RE, van der Loeff MFS, Schuurman R, Hoepelman AI, Wensing AM (2010). Virological follow-up of adult patients in antiretroviral treatment programmes in sub-Saharan Africa: a systematic review. Lancet Infect Dis.

[7] McMahon JH, Elliott JH, Bertagnolio S, Kubiak R, Jordan MR. Viral suppression after 12 months of antiretroviral therapy in low-and middle-income countries: a systematic review. Bull World Health Organ. 2013;91(5):377–85. 10.2471/BLT.12.112946 PMC3646348 23678201

[8] Keiser O, Tweya H, Boulle A, Braitstein P, Schechter M, Brinkhof MW, et al. Switching to second-line antiretroviral therapy in resource-limited settings: comparison of programmes with and without viral load monitoring. AIDS (London, England). 2009;23(14):1867. 10.1097/QAD.0b013e32832e05b2 PMC2956749 19531928

[9] Braun A, Sekaggya-Wiltshire C, Scherrer AU, Magambo B, Kambugu A, Fehr J (2017). Early virological failure and HIV drug resistance in Ugandan adults co-infected with tuberculosis. AIDS Res Ther.

[10] Abdissa A, Yilma D, Fonager J, Audelin AM, Christensen LH, Olsen MF (2014). Drug resistance in HIV patients with virological failure or slow virological response to antiretroviral therapy in Ethiopia. BMC Infect Dis.

[11] Castilla J, Del Romero J, Hernando V, Marincovich B, García S, Rodríguez C (2005). Effectiveness of highly active antiretroviral therapy in reducing heterosexual transmission of HIV. JAIDS J Acquir Immune Defic Syndr.

[12] Matthews GV, Sabin CA, Mandalia S, Lampe F, Phillips AN, Nelson MR (2002). Virological suppression at 6 months is related to choice of initial regimen in antiretroviral-naive patients: a cohort study. AIDS.

[13] Quirk E, McLeod H, Powderly W (2004). The pharmacogenetics of antiretroviral therapy: a review of studies to date. Clin Infect Dis.

[14] Haile D, Takele A, Gashaw K, Demelash H, Nigatu D (2016). Predictors of treatment failure among adult antiretroviral treatment (ART) clients in bale zone hospitals, south eastern Ethiopia. PLoS One.

[15] Pillay P, Ford N, Shubber Z, Ferrand RA (2013). Outcomes for efavirenz versus nevirapine-containing regimens for treatment of HIV-1 infection: a systematic review and meta-analysis. PLoS One.

[16] Oette M, Kroidl A, Göbels K, Stabbert A, Menge M, Sagir A (2006). Predictors of short-term success of antiretroviral therapy in HIV infection. J Antimicrob Chemother.

[17] Izudi J, Alioni S, Kerukadho E, Ndungutse D (2016). Virological failure reduced with HIV-serostatus disclosure, extra baseline weight and rising CD4 cells among HIV-positive adults in northwestern Uganda. BMC Infect Dis.

[18] Bienczak A, Denti P, Cook A, Wiesner L, Mulenga V, Kityo C (2016). Plasma efavirenz exposure, sex, and age predict virological response in HIV-infected African children. J Acquir Immune Defic Syndr (1999).

[19] Marzolini C, Telenti A, Decosterd LA, Greub G, Biollaz J, Buclin T (2001). Efavirenz plasma levels can predict treatment failure and central nervous system side effects in HIV-1-infected patients. AIDS.

[20] Revell AD, Alvarez-Uria G, Wang D, Pozniak A, Montaner JS, Lane HC (2013). Potential impact of a free online HIV treatment response prediction system for reducing virological failures and drug costs after antiretroviral therapy failure in a resource-limited setting. Biomed Res Int.

[21] Wang D, Larder B, Revell A, Montaner J, Harrigan R, De Wolf F (2009). A comparison of three computational modelling methods for the prediction of virological response to combination HIV therapy. Artif Intell Med.

[22] Larder B, Wang D, Revell A, Montaner J, Harrigan R, De Wolf F (2007). The development of artificial neural networks to predict virological response to combination HIV therapy. Antivir Ther.

[23] Zazzi M, Incardona F, Rosen-Zvi M, Prosperi M, Lengauer T, Altmann A (2012). Predicting response to antiretroviral treatment by machine learning: the EuResist project. Intervirology.

[24] Revell A, Khabo P, Ledwaba L, Emery S, Wang D, Wood R (2016). Computational models as predictors of HIV treatment outcomes for the Phidisa cohort in South Africa. South Afr J HIV Med.

[25] Revell AD, Wang D, Wood R, Morrow C, Tempelman H, Hamers RL (2013). Computational models can predict response to HIV therapy without a genotype and may reduce treatment failure in different resource-limited settings. J Antimicrob Chemother.

[26] Revell AD, Wang D, Wood R, Morrow C, Tempelman H, Hamers RL (2016). An update to the HIV-TRePS system: the development and evaluation of new global and local computational models to predict HIV treatment outcomes, with or without a genotype. J Antimicrob Chemother.

[27] Revell AD, Ene L, Duiculescu D, Wang D, Youle M, Pozniak A (2012). The use of computational models to predict response to HIV therapy for clinical cases in Romania. Germs.

[28] Castelnuovo B, Kiragga A, Afayo V, Ncube M, Orama R, Magero S (2012). Implementation of provider-based electronic medical records and improvement of the quality of data in a large HIV program in sub-Saharan Africa. PLoS One.

[29] Kamya MR, Mayanja-Kizza H, Kambugu A, Bakeera-Kitaka S, Semitala F, Mwebaze-Songa P (2007). Predictors of long-term viral failure among ugandan children and adults treated with antiretroviral therapy. JAIDS J Acquir Immune Defic Syndr..

[30] Castelnuovo B, Kiragga A, Mubiru F, Kambugu A, Kamya M, Reynolds SJ (2016). First-line antiretroviral therapy durability in a 10-year cohort of naïve adults started on treatment in Uganda. J Int AIDS Soc.

[31] Castelnuovo B, Kiragga A, Musaazi J, Sempa J, Mubiru F, Wanyama J (2015). Outcomes in a cohort of patients started on antiretroviral treatment and followed up for a decade in an Urban Clinic in Uganda. PLoS One.

[32] Mukonzo JK (2011). Pharmacokinetic aspects of HIV/AIDS, Tuberculosis and Malaria: Emphasis on the Ugandan population [PhD].

[33] Yu C-N, Greiner R, Lin H-C, Baracos V, editors. Learning patient-specific cancer survival distributions as a sequence of dependent regressors. Adv Neural Inf Proces Syst; 2011.

[34] Langford SE, Ananworanich J, Cooper DA (2007). Predictors of disease progression in HIV infection: a review. AIDS Res Ther.

[35] Vergouwe Y, Steyerberg EW, Eijkemans MJ, Habbema JDF (2005). Substantial effective sample sizes were required for external validation studies of predictive logistic regression models. J Clin Epidemiol.

[36] Riley RD, Ensor J, Snell KI, Debray TP, Altman DG, Moons KG (2016). External validation of clinical prediction models using big datasets from e-health records or IPD meta-analysis: opportunities and challenges. BMJ.

[37] Nash JC. Compact numerical methods for computers: linear algebra and function minimisation. Boca Raton: CRC Press; 1990.

[38] Dai Y-H. A perfect example for the BFGS method. Math Program. 2013;138(1–2):501–30.

[39] Broyden CG (1970). The convergence of a class of double-rank minimization algorithms 2. The new algorithm. IMA J Appl Math.

[40] Efron B (1982). The jackknife, the bootstrap, and other resampling plans.

[41] Steyerberg EW, Vickers AJ, Cook NR, Gerds T, Gonen M, Obuchowski N (2010). Assessing the performance of prediction models: a framework for some traditional and novel measures. Epidemiology (Cambridge, Mass).

[42] Austin PC, Steyerberg EW (2014). Graphical assessment of internal and external calibration of logistic regression models by using loess smoothers. Stat Med.

[43] DeGroot MH, Fienberg SE (1983). The comparison and evaluation of forecasters. The statistician.

[44] Steyerberg EW, Vergouwe Y (2014). Towards better clinical prediction models: seven steps for development and an ABCD for validation. Eur Heart J.

[45] Brier GW (1950). Verification of forecasts expressed in terms of probability. Mon Weather Rev.

[46] Lasko TA, Bhagwat JG, Zou KH, Ohno-Machado L (2005). The use of receiver operating characteristic curves in biomedical informatics. J Biomed Inform.

[47] Hanley JA, McNeil BJ (1982). The meaning and use of the area under a receiver operating characteristic (ROC) curve. Radiology.

[48] Powers DM (2011). Evaluation: from precision, recall and F-measure to ROC, informedness, markedness and correlation.

[49] Youden WJ (1950). Index for rating diagnostic tests. Cancer.

[50] Greiner M, Pfeiffer D, Smith R (2000). Principles and practical application of the receiver-operating characteristic analysis for diagnostic tests. Prev Vet Med.

[51] Fluss R, Faraggi D, Reiser B (2005). Estimation of the Youden index and its associated cutoff point. Biom J.

[52] König IR, Malley J, Weimar C, Diener HC, Ziegler A (2007). Practical experiences on the necessity of external validation. Stat Med.

[53] Singal AG, Mukherjee A, Elmunzer BJ, Higgins PD, Lok AS, Zhu J (2013). Machine learning algorithms outperform conventional regression models in predicting development of hepatocellular carcinoma. Am J Gastroenterol.

[54] Goldstein BA, Navar AM, Carter RE (2016). Moving beyond regression techniques in cardiovascular risk prediction: applying machine learning to address analytic challenges. Eur Heart J.

[55] Caruana R. Multitask learning: Learning to learn. Norwell: Kluwer Academic Publishers; 1998. p. 95–133.

[56] Wiens J, Guttag J, Horvitz E (2016). Patient risk stratification with time-varying parameters: a multitask learning approach. J Mach Learn Res.

[57] Singh A, Nadkarni G, Gottesman O, Ellis SB, Bottinger EP, Guttag JV (2015). Incorporating temporal EHR data in predictive models for risk stratification of renal function deterioration. J Biomed Inform.

[58] Dhoro M. CYP2B6*6 screening; potential benefits and challenges in HIV therapy in Sub-Saharan Africa. J Clin Cell Immunol. 2017;8(2):491.

[59] JUNPo HIV/AIDS (2013). Access to antiretroviral therapy in Africa: status report on progress towards the 2015 targets. Geneva.

[60] Wanyenze RK, Wagner G, Alamo S, Amanyire G, Ouma J, Kwarisima D, et al. Evaluation of the efficiency of patient flow at three HIV clinics in Uganda. AIDS Patient Care STDs. 2010;24(7):441–6. 10.1089/apc.2009.0328 PMC2933556 20578908

